# Remote-Sensing Image Classification Based on an Improved Probabilistic Neural Network

**DOI:** 10.3390/s90907516

**Published:** 2009-09-23

**Authors:** Yudong Zhang, Lenan Wu, Nabil Neggaz, Shuihua Wang, Geng Wei

**Affiliations:** 1 School of Information Science and Engineering, Southeast University, Nanjing 210009, China; E-Mails: shuihuaw2007@gmail.com (S.W.); wei_geng@163.com (G.W.); 2 Signal-Image-Parole Laboratory, Department of Computer Science, University of Science and Technology – Oran, Oran, Algeria; E-Mail: neggaz_nabil@yahoo.fr

**Keywords:** polarimetric SAR, Probabilistic neural network, gray-level co-occurrence matrix, principle component analysis, Brent’s Search

## Abstract

This paper proposes a hybrid classifier for polarimetric SAR images. The feature sets consist of span image, the H/A/α decomposition, and the GLCM-based texture features. Then, a probabilistic neural network (PNN) was adopted for classification, and a novel algorithm proposed to enhance its performance. Principle component analysis (PCA) was chosen to reduce feature dimensions, random division to reduce the number of neurons, and Brent’s search (BS) to find the optimal bias values. The results on San Francisco and Flevoland sites are compared to that using a 3-layer BPNN to demonstrate the validity of our algorithm in terms of confusion matrix and overall accuracy. In addition, the importance of each improvement of the algorithm was proven.

## 1. Introduction

The classification of different objects, as well as different terrain characteristics, with single channel monopolarisation SAR images can carry a significant amount of error, even when operating after multilooking [1]. One of the most challenging applications of polarimetry in remote sensing is landcover classification using fully polarimetric SAR (PolSAR) images.

The Wishart maximum likelihood (WML) method has often been used for PolSAR classification [2]. This method uses the amplitudes of the elements in the covariance or coherency matrices. However, it does not explicitly take into consideration the phase information within polarimetric data, which plays a direct role in the characterization of a broad range of scattering processes. Furthermore, the covariance or coherency matrices are determined after spatial averaging and therefore can describe only stochastic scattering processes, while certain objects, such as man-made objects, are better characterized at a pixel-level [3].

To overcome above shortcomings, polarimetric decompositions were introduced with an aim to establish a correspondence between the physical characteristics of the considered areas and the observed scattering mechanisms. There are seven famous decomposition methods: Pauli [4], Krogager [5], Freeman [6], Huynen [7], Barnes [8], Cloude [9] and Holm [8]. The most effective method among these is the Cloude decomposition, also known as the H/A/α method.

Recently, texture information has been extracted and used as a parameter to enhance the classification results. The texture parameters can be defined as many types, such as entropy [10], fractal dimension [11], lacunarity [12], wavelet energy [13], semivariograms [14], and gray-level co-occurrence matrix [15]. Particularly, the gray-level co-occurrence matrices (GLCM) were already successfully applied to classification problems.

Thus, we chose the combination of H/A/α and GLCM as the parameter set of our method. The next problem is how to choose the best classifier. In the past, standard multi-layered feed-forward NNs with a back propagation (BP) algorithm have been applied for SAR image classification [16]. BPs are effective methods since they do not involve complex models and equations as compared to traditional regression analysis. In addition, they can easily adapt to new data through a re-training process.

However, BP needs much effort to determine the architecture of networks and more computations for training. Moreover, BP yields deterministic but not probabilistic results. This makes it technically impractical in classifications. Probabilistic neural networks (PNNs), therefore, are effective alternatives that are faster in determining the network architecture and in training. Moreover, PNNs provide probabilistic viewpoints and deterministic classification results [17].

The input weights and layer weights of PNN can be set directly from the available data, while the bias traditionally is difficult to determine, so it is usually obtained manually either by iterative experiments or by an exhaustive algorithm [18]. In this paper we propose a novel weights/biases setting method. Available input/target pairs are divided into training and validation subsets to reduce the number of neurons, and Brent’s method [19] is adopted to find the optimal biases values since the problem can be regarded as a 1-D interval location problem. In addition, Principal Component Analysis (PCA) is employed [20] in order to reduce the feature dimensions and computation time.

The structure of this paper is as follows: In the next section, we introduce the concept of Pauli decomposition. Section 3 presents the feature set, namely, the span image, the H/A/α decomposition, and the feature derived from GLCM. In section 4, the mechanism, structure and shortcomings of PNNs are introduced. Section 5 proposes our method and expatiates on the three important improvements: PCA, random division and optimization by Brent’s Search. Section 6 applied our method to terrain classification on San Francisco site, and find that our method performs better than 3-layer BPNN method. Section 7 applied our method to crop classification on Flevoland site. Section 8 discusses the significances of combined feature sets, random division, and PCA. Finally, Section 9 concludes this paper.

## 2. Pauli Decomposition

### 2.1. Basic Introduction

The features are derived from the multilook coherence matrix of the polarimetric SAR data. Suppose *S* stands for the measured scattering matrix:

(1) $$S=\left[\begin{array}{ll} S_{\mathrm{hh}} & S_{\mathrm{hv}}\\ S_{\mathrm{vh}} & S_{\mathrm{vv}} \end{array}\right]=\left[\begin{array}{ll} S_{\mathrm{hh}} & S_{\mathrm{hv}}\\ S_{\mathrm{hv}} & S_{\mathrm{vv}} \end{array}\right]$$

where *S_qp_* represents the scattering coefficients of the targets, *p* the polarization of the incident field, *q* the polarization of the scattered field. *S_hv_* equals to *S_vh_* since reciprocity applies in a monostatic system configuration.

The Pauli decomposition expresses the scattering matrix *S* in the so-called Pauli basis, which is given by the following three 2×2 matrices:

(2) $$S_{a}=\frac{1}{\sqrt{2}}\;\left[\begin{array}{ll} 1 & 0\\ 0 & 1 \end{array}\right],\;S_{b}=\frac{1}{\sqrt{2}}\;\left[\begin{array}{ll} 1 & 0\\ 0 & -1 \end{array}\right],\;S_{c}=\frac{1}{\sqrt{2}}\;\left[\begin{array}{ll} 0 & 1\\ 1 & 0 \end{array}\right]$$

Thus, *S* can be expressed as:

(3) $$S={\mathrm{aS}}_{a}+{\mathrm{bS}}_{b}+{\mathrm{cS}}_{c}$$

where:

(4) $$a=\frac{S_{\mathrm{hh}}+S_{\mathrm{vv}}}{\sqrt{2}},\;b=\frac{S_{\mathrm{hh}}+S_{\mathrm{vv}}}{\sqrt{2}},\;c=\sqrt{2}S_{\mathrm{hv}}$$

An RGB image could be formed with the intensities |*a*|^2^, |*b*|^2^, |*c*|^2^. The meanings of *S_a_*, *S_b_*, and *S_c_* are listed in Table 1.

### 2.2. Coherence Matrix

The coherence matrix is obtained as:

(5) $$T=[a,b,c]\;{\left[a,b,c\right]}^{T}=\left[\begin{array}{ccc} T_{11} & T_{12} & T_{13}\\ T_{12}^{*} & T_{22} & T_{23}\\ T_{13}^{*} & T_{23}^{*} & T_{33} \end{array}\right]$$

The average of multiple single-look coherence matrices is the multi-look coherence matrix. (*T*_11_,*T*_22_,*T*_33_) usually are regarded the channels of the polarimetric SAR images.

## 3. Feature Extraction

The proposed features can be divided into three types, which are explained below.

### 3.1. Span

The span or total scattered power indicates the received power by a fully polarimetric system and is given by:

(6) $$M={\left|S_{\mathrm{hh}}\right|}^{2}+{\left|S_{\mathrm{vv}}\right|}^{2}+2{\left|S_{\mathrm{hv}}\right|}^{2}$$

### 3.2. H/A/Alpha Decomposition

Cloude and Potter [9] proposed an algorithm to identify in an unsupervised way polarimetric scattering mechanisms in the *H*-*α* plane. The method extends the two assumptions of traditional ways: 1) azimuthally symmetric targets; 2) equal minor eigenvalues *λ*_2_ and *λ*_3_.

*T* can be rewritten as:

(7) $$T=U_{3}\;\left[\begin{array}{ccc} \lambda_{1} & 0 & 0\\ 0 & \lambda_{2} & 0\\ 0 & 0 & \lambda_{3} \end{array}\right]\;U_{3}^{H}$$

(8) $$U_{3}=\left[\begin{array}{ccc} \text{cos}\;\alpha_{1} & \text{cos}\;\alpha_{2} & \text{cos}\;\alpha_{3}\\ \text{sin}\;\alpha_{1}\;\text{cos}\;\beta_{1}\text{exp}\left(i\delta_{1}\right) & \text{sin}\;\alpha_{2}\;\text{cos}\;\beta_{2}\;\text{exp}\left(i\delta_{2}\right) & \text{sin}\;\alpha_{3}\;\text{cos}\;\beta_{3}\;\text{exp}\left(i\delta_{3}\right)\\ \text{sin}\;\alpha_{1}\;\text{sin}\;\beta_{1}\;\text{exp}\left(i\gamma_{1}\right) & \text{sin}\;\alpha_{2}\;\text{sin}\;\beta_{2}\;\text{exp}\left(i\gamma_{2}\right) & \text{sin}\;\alpha_{3}\;\text{sin}\;\beta_{3}\;\text{exp}\left(i\gamma_{3}\right) \end{array}\right]$$

Then, the pseudo-probabilities of the *T* matrix expansion elements are defined as:

(9) $$P_{i}=\frac{\lambda_{j}}{\sum_{j=1}^{3}\;\lambda_{j}}$$

The entropy indicates the degree of statistical disorder of the scattering phenomenon. It can be defined as:

(10) $$H=\sum_{i=1}^{3}\;-P_{i}\;{\text{log}}_{3}\;P_{i}\;\;0\leq H\leq 1$$

For high entropy values, a complementary parameter (anisotropy) is necessary to fully characterize the set of probabilities. The anisotropy is defined as the relative importance of the second scattering mechanisms [21]:

(11) $$A=\frac{P_{2}-P_{3}}{P_{2}+P_{3}}\;\;\;0\leq A\leq 1$$

The four estimates of the angles are easily evaluated as:

(12) $$\left[\bar{\alpha },\;\bar{\beta },\;\bar{\delta },\;\bar{\gamma }]=\sum_{i=1}^{3}\;P_{i}[\alpha ,\beta ,\delta ,\gamma \right]$$

Thus, vectors from coherence matrix can be represented as (*H*, *A*, *ᾱ*, *β̄*, *δ̄*, *γ̄*).

### 3.3. Texture Features

The Gray level co-occurrence matrix (GLCM) is a text descriptor which takes into account the specific position of a pixel relative to another. The GLCM is a matrix whose elements correspond to the relative frequency of occurrence of pairs of gray level values of pixels separated by a certain distance in a given direction [22]. Formally, the elements of a GLCM *G*(*i*,*j*) for a displacement vector (*a*,*b*) is defined as

(13) G(i, j)=|{(x,y),(t,v):I(r,s)=i&I(t,v)=j}|

Where (*t*,*v*) = (*x*+*a*, *y*+*b*), and |•| is the cardinality of a set. The displacement vector (*a*,*b*) can be rewritten as (*d*, *θ*) in polar coordinates.

GLCMs are suggested to calculate from four displacement vectors with *d* = 1 and *θ* = 0°, 45°, 90°, and 135° respectively. In this study, the (*a*,*b*) are chosen as (0,1), (−1,1), (−1,0), and (−1,−1) respectively, and the corresponding GLCMs are averaged.

The four features are extracted from normalized GLCMs, the sum of which is equal to 1. Suppose the normalized GLCM value at (*i*,*j*) is *p*(*i*,*j*), and their detailed definition are listed in Table 2.

### 3.4. Total Features

The texture features consist of 4 GLCM-based features, which should be multiplied by 3 since there exist three channels (*T*_11_,*T*_22_,*T*_33_). In addition, there are one span feature, and six *H*/*α* parameters. In all, the total features are 1 + 6 + 4 × 3 = 19.

## 4. Probabilistic NN

### 4.1. Mechanism of PNN

Neural networks are widely used in pattern classification since they do not need any information about the probability distribution and the *a priori* probabilities of different classes. PNNs are basically pattern classifiers. They combine the well known Bayes decision strategy with the Parzen non-parametric estimator of the probability density functions (PDF) of different classes. PNNs have been of interest because they yield a probabilistic output and are easy to implement.

Taking a two categories situation as an example, we should decide the known state of nature *θ* to be either *θ*_A_ or *θ*_B_. Suppose a set of measurements is obtained as *p*-dimensional vector *x* = [*x*_1_, …, *x_p_*], the Bayes decision rule becomes:

(14) $$d(x)=\{\begin{array}{ll} \theta_{A} & \text{if}\;h_{A}l_{A}f_{A}(x)>h_{B}l_{B}f_{B}(x)\\ \theta_{B} & \text{if}\;h_{A}l_{A}f_{A}(x)<h_{B}l_{B}f_{B}(x) \end{array}$$

Here, *f*_A_(*x*) and *f*_B_(*x*) are the PDF for categories A and B, respectively. *l*_A_ is the loss function associated with the wrong decision d(*x*) = *θ*_B_ when *θ* = *θ*_A_, *l*_B_ is the loss function associated with the wrong decision d(*x*) = *θ*_A_ when *θ* = *θ*_B_, and the losses associated with correct decisions are taken to be zero. *h*_A_ and *h*_B_ are the *a priori* probability of occurrence of patters from category A and B, respectively.

In a simple case that assumes the loss function and *a priori* probability are equal, the Bayes rule classifies an input pattern to the class with higher PDF. Therefore, the accuracy of the decision boundaries depends on what the underlying PDFs are estimated. Parzen’s results can be extended to estimate in the special case where the multivariate kernel is a product of univariate kernels. In the particular case of the Gaussian kernel, the multivariate estimates can be expressed as:

(15) $$f_{A}(x)=\frac{1}{{\left(2\pi \right)}^{p/2}\sigma^{p}}\frac{1}{m}\sum_{i=1}^{m}\text{exp}\left[-\frac{{\left(x-x_{Ai}\right)}^{T}\left(x-x_{Ai}\right)}{{2\sigma }^{2}}\right]$$

Here, *m* is the number of training vectors in category A, p is the dimensionality of the training vectors, *x*_A*i*_ is the *i*th training vector for category A, and *σ* is the smoothing parameter. It should be noted that *f*_A_(*x*) is the sum of small multivariate Gaussian distributions centered at each training sample, but the sum is not limited to being Gaussian.

### 4.2. PNN Structure

**Figure 1.**
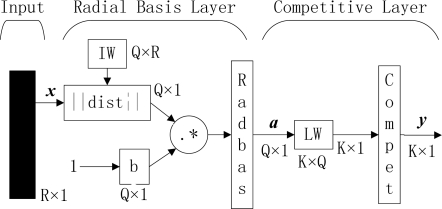
Outline of PNN (R, Q, and K represent number of elements in input vector, input/target pairs, and classes of input data, respectively. IW and LW represent input weight and layer weight, respectively).

Figure 1 shows the outline of PNN. When an input is presented, the first layer computes distances from the input vector to the input weights (IW), and produces a vector whose elements indicate how close the input is to the IW. The second layer sums these contributions for each class of inputs to produce as its net output a vector of probabilities. Finally, a *compet* transfer function on the output of the second layer picks the maximum of these probabilities, and produces a 1 for that class and a 0 for other classes.

The mathematical expression of PNN can be expressed as:

(16) a=radbas(‖IW−x‖•b)

(17) y=compet(LW•a)

In this paper, the *radbas* is selected as:

(18) $$\text{radbas}(n)=\text{exp}({-n}^{2})$$

The *compet* function is defined as:

(19) $$\text{compet}(n)=e_{i}=\left[0\;0\;\cdots \;\;0\;\underset{i}{1}\;0\;\;\cdots \;0\right],\;\;n(i)=\text{max}(n)$$

This type of setting can produce a network with zero errors on training vectors, and obviously it does not need any training.

### 4.3. Shortcomings of Traditional PNN

Suppose *P* and *T* denote the set of training vector *x* and corresponding target vector *y*, namely, *P* = [*x*_1_, *x*_2_, …*x_Q_*], and *T* = [*y*_1_, *y*_2_, *y_Q_*]. IW and LW are set traditionally as follows:

(20) IW=P

(21) LW=T

However, it is obvious that *Q* is usually very large, and then the net will be too big and consume too much computation time. On the other hand, to simplify the setting of bias *b*, all of its components are considered as equal [23]. Even so, the setting of *b* is still a challenge. Although errors on training vectors are always zero, the errors on test vectors are greatly dependent with the value of *b*.

If it is too small, the spread of each radial basis layer function becomes too large, and the network will take too many nearby design vectors into account, moreover, the radial basis neurons will output large values (near 1) for all the inputs used to design the network. If it is too larger, the spread becomes near zero, and the network will degrades as a nearest neighbor classifier.

## 5. A Novel Method of Weights/Biases Setting

**Figure 2.**
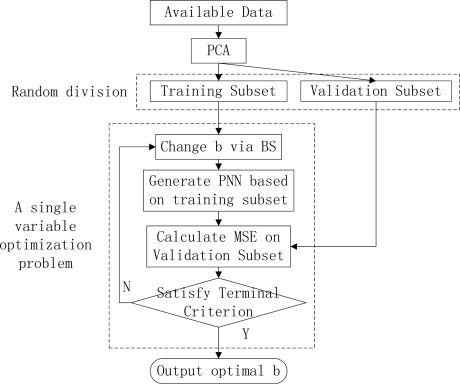
The outline of our method.

Here we propose a novel method to solve the above two problems. The main idea is shown in Figure 2. Our improvement lies in the PCA, the random division, and the single variable optimization.

### 5.1. Feature Reduction

Excessive features increase computation times and storage memory. Furthermore, they sometimes make classification more complicated, which is called the curse of dimensionality. It is necessary to reduce the number of features.

Principal component analysis (PCA) is an efficient tool to reduce the dimensionality of a data set consisting of a large number of interrelated variables, while retaining most of the variations. It is achieved by transforming the data set to a new set of ordered variables. This technique has three effects: it orthogonalizes the components of the input vectors so that uncorrelated with each other, it orders the resulting orthogonal components so that those with the largest variation come first, and eliminates those components contributing the least to the variation in the data set.

**Figure 3.**
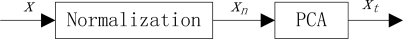
Using normalization before PCA.

It should be noted that the input vectors should be normalized to have zero mean and unity variance before performing PCA, which is shown in Figure 3.

The normalization is a standard procedure. Details about PCA can be found in Ref. [24].

### 5.2. Random Division

Realistic sample numbers *Q* are generally very large, which leads to a quite large PNN. Thus, we divide the available data into two subsets: training subset and validation subset. The ratio of each is called *trainRatio* and *validRatio* respectively. In order to save the storage room of the net and to fasten the computation, the *trainRatio* is set as small as possible, and meanwhile it should not affect the accuracy of the NN.

### 5.3. Optimization by Brent’s Search

The goal of finding optimal *b* can be obtained by solving this problem: find the minimum MSE on validation subset of the corresponding *b*. This can be depicted as a single-variable optimization problem in the dash-line rectangle in Figure 2. Brent’s Search (BS) method is adopted to solve this optimization problem.

BS is a linear search, a hybrid of the golden section search and a quadratic interpolation. Golden section search has a first-order rate of convergence, while polynomial interpolations have an asymptotic rate faster than super-linear. On the other hand, the rate of convergence for the golden section search starts when the algorithm is initialized, whereas the asymptotic behavior for the polynomial interpolations can take many iterations to become apparent. BS attempts to combine the best features of both approaches. BS has the advantage that it does not require computation of the derivative, which greatly fits the optimization problem.

## 6. Terrain Classification

The NASA/JPL AirSAR L-band data for the San Francisco (California, USA) area was used for the experiments. Its size is 1,024 × 900. In order to reduce the computations, the sub-area with size 600 × 600 was extracted from the left-upper point of original image. The ground truth of the test site can be found at Ref. [2].

Quantitative information about the experiment is described as follows, where ‘•’ denotes parameters known before simulation and ‘♦’ denotes the parameters obtained at the initial stage of the experiment.

•Number of features: 19
♦ Number of reduced features by PCA: 11 (obtained by performing PCA on total available pairs)
•Location of Sub San Francisco Area:
  - X-range: 1–600
  - Y-range: 1–600
•Location of Training/Test Rectangular Area (the first and second pixels denote the coordinate of the left-upper point of the rectangle, the third and forth pixels denote the width and length of the rectangle)
  - Sea:
    - Training Area1 [100 500 60 60]
    - Training Area2 [300 200 60 60]
    - Test Area [500 50 60 60]
  - Urban:
    - Training Area1 [450 400 60 60]
    - Training Area2 [500 250 60 60]
    - Test Area [500 530 60 60]
  - Vegetated
    - Training Area1 [50 50 60 60]
    - Training Area2 [50 250 60 60]
    - Test Area [320 450 60 60]
•Parameters of GLCM
  - local area: 5 × 5 (pixels)
  - Number of gray levels: 8
  - Offset: [0 1]
•Properties of available training/target pairs
  - Pairs = 21,600
  - R = 11
  - K = 3
  - P (size 11 × 21,600)
  - T (size 3 × 21,600)
    ♦ Training Ratio: 0.01 (obtained by simple iterative tests)
    •Validation Ratio: 0.99
•Properties of NN optimized by our approach
  •Q = Pairs × trainRatio = 216
  ♦ b = 4.73(obtained by BS method)
  •IW = P (size: 216 × 11)
  •LW = T (size: 3 × 216)
•Properties of BS Method
  - Tolerance X Value: 1e–3
  - Tolerance Function Value: 1e–5
  - Maximum Iterative Steps: 30
•Hardware: Pentium 4 CPU 1.66 GHz, 512 MB of RAM
•Software: PolSARpro v4.0, Neural Network Toolbox of Matlab 7.8(R2009)

### 6.1. Denoising by Lee Filter

**Figure 4.**
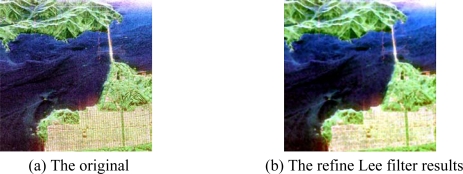
Pauli image of sub-area of San Francisco.

The sub-area (600 × 600) is shown in Figure 4(a). The Refined Lee filter (Window size = 7) is used to reduce the speckle noise and the results are shown in Figure 4(b). The Lee filter adapts the amount of filtering to the local statistics. Homogeneous areas are filtered with the maximum strength where point scatterers are let unfiltered. The refined filter could use directional windows to preserve edges and heterogeneous features [25].

### 6.2. Full Features Set

**Figure 5.**
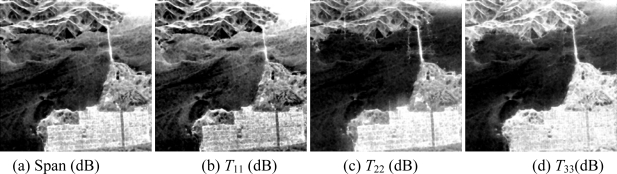
Basic span image and three channels image.

**Figure 6.**
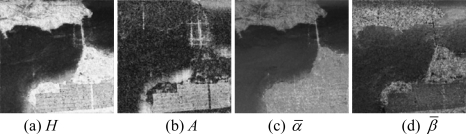

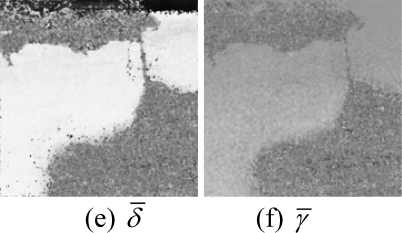
Parameters of H/A/Alpha decomposition.

**Figure 7.**
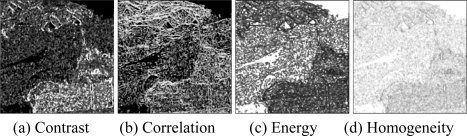
GLCM-based features of *T*_11._

**Figure 8.**
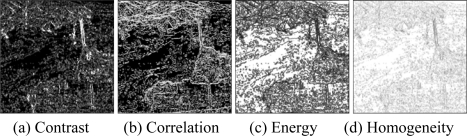
GLCM-based features of *T*_22._

**Figure 9.**
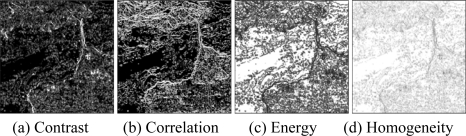
GLCM-based features of *T*_33._

Then, the basic span image and three channels (*T*_11_,*T*_22_,*T*_33_) are easily obtained and shown in Figure 5. The parameters of H/A/Alpha decomposition are shown in Figure 6. The GLCM-based parameters of *T*_11_, *T*_22_, *T*_33_ are shown in Figures 7–9.

### 6.3. Feature Reduction by PCA

**Figure 10.**
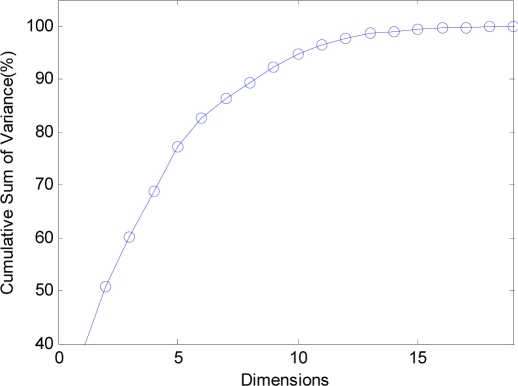
The curve of cumulative sum of variance with dimensions.

The curve of cumulative sum of variance with dimensions of reduced vectors via PCA is shown in Figure 10. The detailed data are listed in Table 3. It shows that only 11 features, half the original features only, could preserve 96.36% of variance.

Thus, 11 new features obtained via PCA are input to the NN for classification training.

### 6.4. Training Preparation

**Figure 11.**
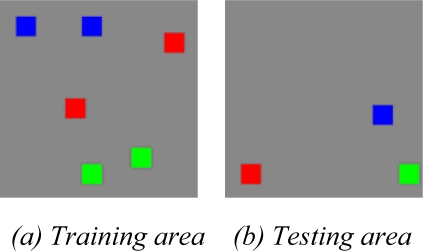
Sample data of San Francisco (Red denotes sea, green urban areas, blue vegetated zones).

The classification is run over three classes, the sea, the urban areas and the vegetated zones. The training and testing areas are selected manually shown in Figures 11(a)–(b), respectively. Each square has a size of 60×60. In total, there are 21,600 pixels for training, and 10,800 pixels for testing. In this experiment, *trainRatio* is adjusted finally as 0.01, namely, the *validRatio* equals 0.99. In this way, the network only has 1% neurons of that constructed by traditional approach. The training subset and validation subset of the training area are divided randomly.

### 6.5. Weights/Biases Setting

**Figure 12.**
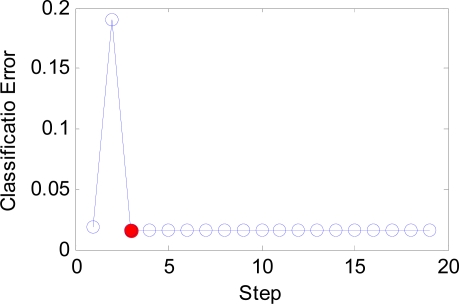
The curve of error versus step.

**Figure 13.**
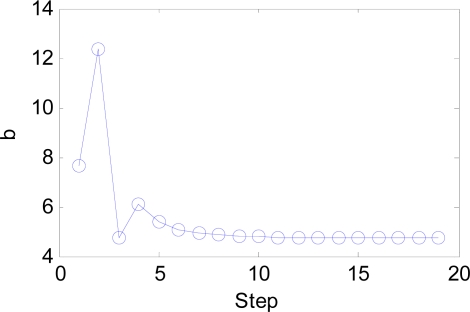
The curve of b versus step.

The IW and LW are easily set according to our novel approach, and the number of neurons decreases from 21,600 to only 216. The *b* is estimated by BS method. Its initial range is set as [0.01, 20], which is large enough to contain the optimal point. The curve of classification error versus the steps is shown in Figure 12. It is evident that the classification error converges at only three steps shown in the red dot. However, BS will continue to search the best *b* value since the tolerance of *b* is set as small as 1e–3. The whole process of the change of b is shown in Figure 13.

The optimal *b* is found as 4.73, with the smallest error 1.557%, namely, the highest classification accuracy 98.44%.

### 6.6. Application to the Whole Image

**Figure 14.**
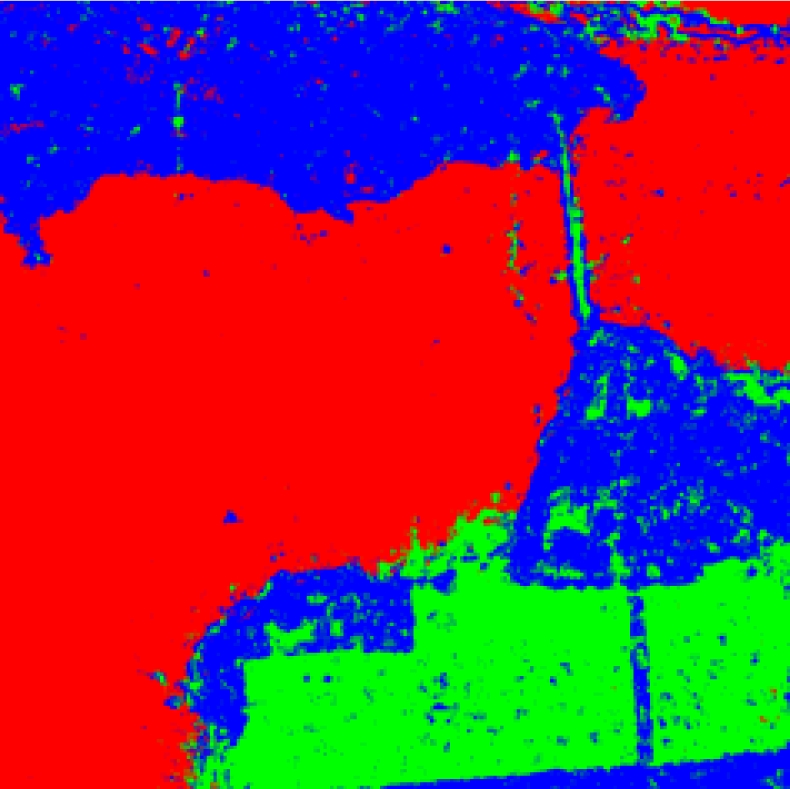
Classification results of the whole image.

We use the trained PNN to classify the whole image, and the results are shown in Figure 14. The brims of length 3 are not calculated considering the local area of GLCM, so the size here is only 594 × 594.

From Figure 14 it makes clear that the sea can be classified perfectly, while the vegetated and urban areas are easily inter-confused. The next section will calculate the confusion matrix which reflects the degree of confusion between the three classes.

### 6.7. Comparison with Other Approaches

Finally, our method is compared to the 3-layer BPNN [16]. The confusion matrices (CM) by each methods on training area and testing area are listed in Table 4. The element of *i*th row and *j*th column in the 3 × 3 matrix represents the amount of pixels belonging to class *j* as user defined are assigned to class *i* after the supervised classification.

It is obvious that the classification accuracies of our proposed method in training area are all higher than 32.5% (33.3% denotes the perfect classification). For the testing area, classification accuracies are all higher than 30.1%. The main drawback is around 3.3% of vegetated zones are misclassified as urban area.

The overall accuracies are calculated as CM_11_ + CM_22_ + CM_33_ and listed in Table 5, that demonstrates our method has a higher overall accuracy in both training area and testing area than those of 3-layer BPNN. The reason our method outperforms the 3-layer BPNN lies in not only the fact that PNN is adept at predicting the probabilistic results, but also the selected features sets are more discernable.

## 7. Crop Classification

Flevoland, an agricultural area in The Netherlands, was chosen as another example. The site is composed of strips of rectangular agricultural fields. The scene is designated as a supersite for the earth observing system (EOS) program, and is continuously surveyed by the authorities. The ground truth of the test site can be seen in Ref [26].

•Number of features: 19
♦ Number of reduced features by PCA: 13 (obtained by performing PCA on total available pairs)
•Location of Train/Test Rectangular Area
  - Bare Soil 1:
    - Train Area [240 300 20 20]
    - Test Area [770 490 20 20]
  - Bare Soil 2
    - Train Area [335 440 20 20]
    - Test Area [420 425 20 20]
  - Barley
    - Train Area [285 500 20 20]
    - Test Area [765 425 20 20]
  - Forest
    - Train Area [959 155 20 20]
    - Test Area [900 490 20 20]
  - Grass
    - Train Area [535 240 20 20]
    - Test Area [500 303 20 20]
  - Lucerne
    - Train Area [550 495 20 20]
    - Test Area [505 550 20 20]
  - Peas
    - Train Area [523 330 20 20]
    - Test Area [436 200 20 20]
  - Potatoes
    - Train Area [32 40 20 20]
    - Test Area [655 307 20 20]
  - Rapeseed
    - Train Area [188 200 20 20]
    - Test Area [280 250 20 20]
  - Stem Beans
    - Train Area [800 350 20 20]
    - Test Area [777 384 20 20]
  - Sugar beet
    - Train Area [877 444 20 20]
    - Test Area [650 225 20 20]
  - Water
    - Train Area [965 50 20 20]
    - Test Area [961 201 20 20]
  - Wheat
    - Train Area [780 710 20 20]
    - Test Area [700 520 20 20]
•Parameters of GLCM
  - local area: 5×5 (pixels)
  - Number of gray levels: 8
  - Offset: [0 1]
•Properties of available training/target pairs
  - Pairs = 5200
  - R = 13
  - K = 13
  - P (size 13 × 5200)
  - T (size 13 × 5200)
    ♦ Training Ratio: 0.2 (obtained by simple iterative tests)
    •Validation Ratio: 0.8
•Properties of NN optimized by our approach
  •Q = Pairs × trainRatio = 1040
  ♦ b = 1.0827(obtained by BS method)
  •IW = P (size: 13 × 1040)
  •LW = T (size: 13 × 1040)
•Properties of BS Method
  - Tolerance X Value: 1e–3
  - Tolerance Function Value: 1e–5
  - Maximum Iterative Steps: 30
•Hardware: Pentium 4 CPU 1.66 GHz, 512 MB of RAM
•Software: PolSARpro v4.0, Neural Network Toolbox of Matlab 7.8(R2009)

### 7.1. Refine Lee Filter

**Figure 15.**
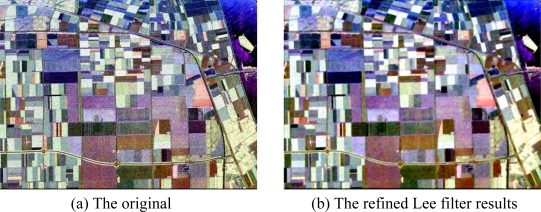
Pauli Image of Flevoland (1024 × 750).

The Pauli image of Flevoland is shown in Figure 15(a), and the refined Lee filtered image (Window Size = 7) is shown in Figure 15(b).

### 7.2. Full Features

**Figure 16.**
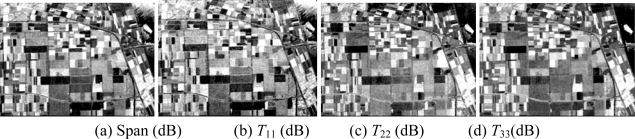
Basic span image and three channels image.

**Figure 17.**
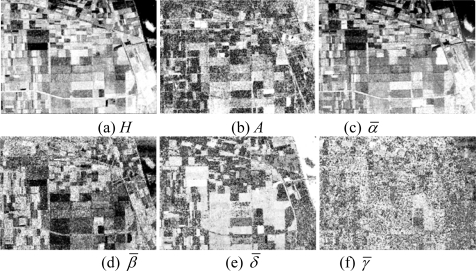
Parameters of H/A/Alpha decomposition.

**Figure 18.**
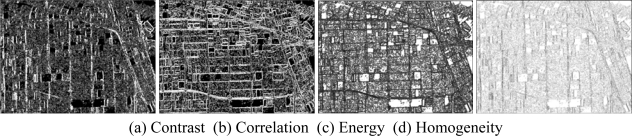
GLCM-based features of T_11_.

**Figure 19.**
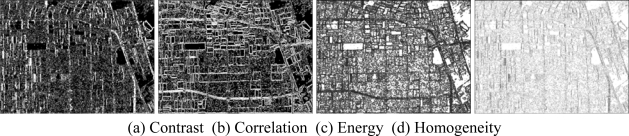
GLCM-based features of T_22_.

**Figure 20.**
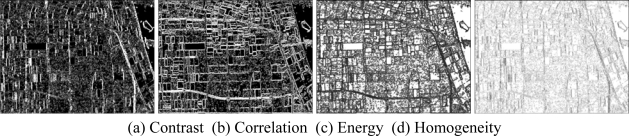
GLCM-based features of T_33_.

The basic span image and three channels (*T*_11_,*T*_22_,*T*_33_) are easily obtained and shown in Figure 16. The parameters of H/A/Alpha decomposition are shown in Figure 17. The GLCM-based parameters of *T*_11_, *T*_22_, *T*_33_ are shown in Figures 18–20.

### 7.3. Feature Reduction

**Figure 21.**
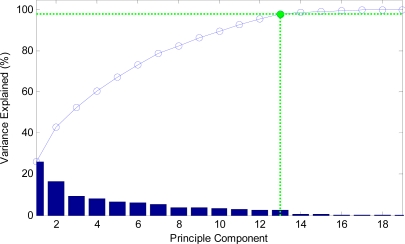
The curve of cumulative sum of variance with dimensions.

The curve of cumulative sum of variance with dimensions of reduced vectors via PCA is shown in Figure 21. The detailed data are listed in Table 6. It shows that only 13 features, which are only half the original features, could preserve 98.06% of variance.

### 7.4. Training Preparation

**Figure 22.**
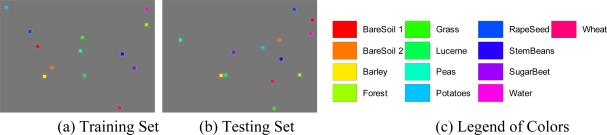
Sample data areas of Flevoland.

The classification is run over 11 classes, bare soil 1, bare soil 2, barley, forest, grass, Lucerne, peas, potatoes, rapeseed, stem beans, and sugar beet. They are selected manually according to the ground truth [26]. The training set and testing set are shown in Figure 22. Each square has a size of 20 × 20. In total, there are 5,200 pixels for training, and 5,200 pixels for testing.

Since the types of classes increase (3 to 13) while the available data decrease (21,800 to 5,200), thus, we should dispose more data to the training subset. Finally, *trainRatio* is adjusted as 0.2, while *validRatio* is set as 0.8. In this way, the network only has 1/5 neurons of that constructed by traditional approach. The training subset and validation subset of the training area are divided randomly.

### 7.5. Weights/Biases Setting

**Figure 23.**
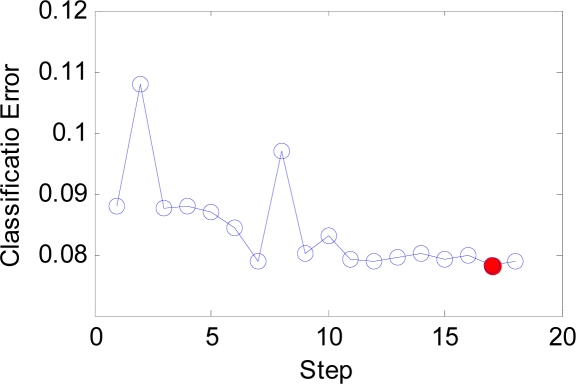
The curve of error versus step.

**Figure 24.**
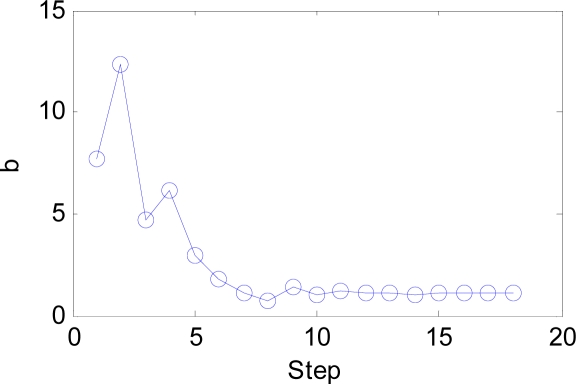
The curve of b versus step.

The IW and LW are easily set according to our novel approach, and the number of neurons decreases from 5200 to only 1040. The *b* is estimated by BS method. Its initial range is set as before. The curve of classification error versus the steps is shown in Figure 23. It is evident that the classification error reaches the minimum at 17th steps shown in the red dot. The whole process of the change of *b* is shown in Figure 24.

The optimal *b* is found as 1.0827, with the smallest error 7.8%, namely, the highest classification accuracy on the validation subset of training area is 98.44%.

### 7.6. Classification Results

**Figure 25.**
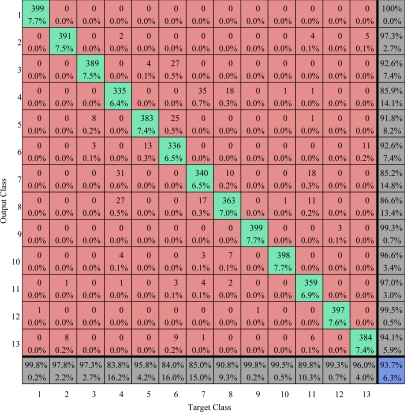
Confusion matrix comparison on train area (values are given in percent) The overall accuracy is 93.71%.

**Figure 26.**
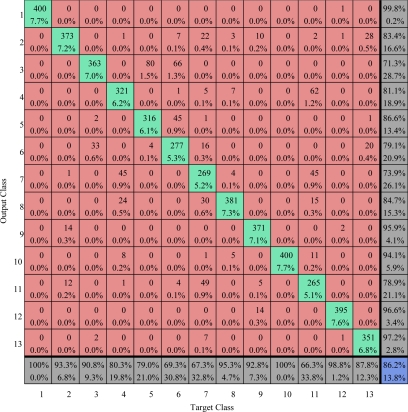
Confusion matrix comparison on test area (values are given in percent) The overall vccuracy is 86.2%.

The confusion matrices on training area and testing area are calculated and listed in Figure 25 and Figure 26. The overall accuracy of our method on training area and test area are 93.71% and 86.2% respectively.

**Figure 27.**
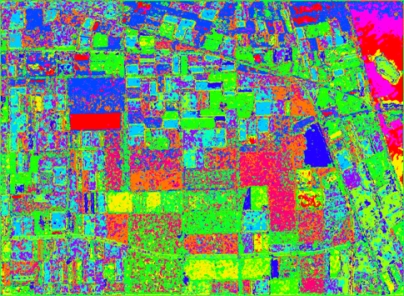
Classification Map of our method.

We apply our method on the whole image. The results are shown in Figure 27. From Figure 27 it is clear that our method can classify most of areas correctly.

## 8. Discussion

The BS has an important effect on our algorithm as shown in Figure 12 and Figure 23. It can guide users to find the optimal *b* value in quite short steps. Otherwise, the users will take a long time with the help of exhaustive search algorithm. The function of the combined feature, random division and PCA will be discussed in detail at following paragraphs.

### 8.1. Single Type of Feature Set versus Combined Feature Sets

The feature sets can be divided into two types. One is the polarimetric feature set, which contains the span, the six H/A/α parameters; the other is the texture feature set, which contains the properties extracted from the GLCM.

Table 7 lists the classification accuracy of classifiers using polarimetric feature set, texture feature set, and combined feature set. It indicates that the polarimetric features contribute most to the classification while the texture feature contribute less. Then, we can find that the combined feature set performs better than each single. Thus, our classifier using combined feature set can be regarded as an feature fusion method.

### 8.2. With and without Random Division

If we do not use the random division, the structure of PNN will increase 1/*trainRatio* times. Consequently, the computation will become a burden with very little improve on classification overall accuracy. Taking the San Francisco area as the example, four square areas of different size are picked out randomly from the image, and are classified by PNNs with and without random division. The computation time and overall accuracy of each are listed in Table 8.

Table 8 indicates that the computation time of traditional method is only 46 times of that of our method for 10 × 10 area, however, the ratio rockets to 516 for 40 × 40 area. Moreover, for a larger size area, such as 50 × 50, it cannot work because of the lack of memory.

From another point of view, the overall accuracy of traditional method was expected to be much higher than that of our method since it uses a great many neurons, whereas, in fact, they are nearly the same. The reason may consist of the optimization of *b* in our method. Accordingly, our method of weights/biases setting is valid and effective, and it is superior to traditional method in terms of computation time and storage room while it can maintain a high overall accuracy.

### 8.3. With and without PCA

**Figure 28.**
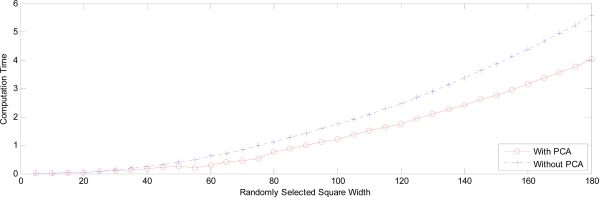
Computation time with square width.

PNNs with and without PCA are investigated in the same manner as in Section 7.1. Their computation times are depicted in Figure 28, which indicates that PNN with PCA enjoys a less computation time than that of PNN without PCA. Their time differences are gradually becoming large as the width of the randomly selected area is increasing.

In addition, the overall accuracies of these two PNNs are observed. It should be noted that input data of the PNN without PCA still should be normalized although the PCA is omitted, otherwise the performance of PNN will decrease rapidly.

**Figure 29.**
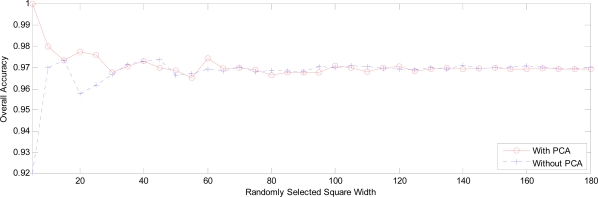
The overall accuracy versus square width.

The overall accuracies obtained by the two PNNs are pictured in Figure 29. It demonstrates that the PNN with PCA outperforms PNN without PCA on the small test area (width < 40). As the area becomes large (40 < width < 47), the PNN without PCA is better. Finally, as the area becomes large enough (width > 47), these performances of the two PNNs are nearly equivalent. Therefore, our method embedding PCA can performs faster, and has no loss of overall accuracy.

## 9. Conclusions

In this paper, a hybrid feature set has been introduced which is made up of the span image, the H/A/α decomposition, and the GLCM-based texture features. Then, a probabilistic neural network has been established. We proposed a novel weights/biases setting method based on Brent’s method and PCA. The method can decrease the feature set, reduce the number of neurons, and find optimal bias values.

Experiments of terrain classification on a San Francisco site and a crop classification on Flevoland show that our method can obtain good results which are more accurate than those of 3-layer BPNN. Afterwards, combined feature set, random division and PCA are assumed to be omitted in turn, and the results prove the indispensability of each improvement.

## Figures and Tables

**Table 1.** Pauli bases and their corresponding meanings.

**Pauli Bases**	**Meaning**
S_a_	Single- or odd-bounce scattering
S_b_	Double- or even-bounce scattering
S_c_	Those scatterers which are able to return the orthogonal polarization to the one of the incident wave (forest canopy)

**Table 2.** Properties of GLCM.

**Property**	**Description**	**Formula**
Contrast	Intensity contrast between a pixel and its neighbor	$\sum_{i,j}\left|i-j{|}^{2}p(i,j\right)$
Correlation	Correlation between a pixel and its neighbor (μ denotes the expected value, and σ the standard variance)	$\sum_{i,j}\frac{\left(i-\mu_{i}\right)\left(j-\mu_{j}\right)p(i,j)}{\sigma_{i}\sigma_{j}}$
Energy	Energy of the whole image	$\sum_{i,j}p{\left(i,j\right)}^{2}$
Homogeneity	Closeness of the distribution of GLCM to the diagonal	$\sum_{i,j}\frac{p(i,j)}{1+|i-j|}$

**Table 3.** Detailed data of PCA on 19 features.

**Dimensions**	1	2	3	4	5	6	7	8	9
**Variance (%)**	37.97	50.81	60.21	68.78	77.28	82.75	86.27	89.30	92.27

**Dimensions**	10	11	12	13	14	15	16	17	18
**Variance (%)**	94.63	96.36	97.81	98.60	99.02	99.37	99.62	99.80	99.92

**Table 4.** Comparison of confusion matrix. (O denotes the output class, T denotes the target class).

		**Training Area**			**Testing Area**		
		**Sea(T)**	**Urb(T)**	**Veg(T)**	**Sea(T)**	**Urb(T)**	**Veg(T)**

**3-layer BPNN**	**Sea(O)**	7158	4	60	3600	42	5
		33.1%	0.0%	0.3%	33.3%	0.4%	0.0%
	**Urb(O)**	0	6882	136	0	3429	355
		0%	31.9%	0.6%	0.0%	31.7%	3.3%
	**Veg(O)**	42	314	7004	0	129	3240
		0.2%	1.4%	32.4%	0.0%	1.2%	30.0%

**Our Method**	**Sea(O)**	7150	0	76	3597	33	0
		33.1%	0.0%	0.4%	33.3%	0.3%	0.0%
	**Urb(O)**	2	7074	74	0	3445	354
		0%	32.8%	0.3%	0.0%	31.9%	3.3%
	**Veg(O)**	48	126	7050	3	122	3246
		0.2%	0.6%	32.6%	0.0%	1.1%	30.1%

**Table 5.** Overall accuracies (values are given in percent).

	**Training Area**	**Testing Area**
**3-layer BPNN**	97.4%	95.1%
**Our Method**	98.5%	95.3%

**Table 6.** Detailed data of PCA on 19 features.

**Dimensions**	1	2	3	4	5	6	7	8	9
**Variance (%)**	26.31	42.98	52.38	60.50	67.28	73.27	78.74	82.61	86.25

**Dimensions**	10	11	12	**13**	14	15	16	17	18
**Variance (%)**	89.52	92.72	95.50	**98.06**	98.79	99.24	99.63	99.94	99.97

**Table 7.** Comparison of PNNs using polarimetric feature set, texture feature set, and combined feature set (TR denotes Classification Accuracy of Total Random).

**Site**		**Polarimetric feature set**	**Texture feature set**	**Combined feature set**
***San Francisco (TR=33.3%)***	***Training Area***	*97.1%*	*59.9%*	*98.5%*
	***Test Area***	*87.4%*	*45.9%*	*95.3%*
***Flevoland (TR=7.69%)***	***Training Area***	*92.2%*	*48.0%*	*93.7%*
	***Test Area***	*72.2%*	*24.1%*	*86.2%*

**Table 8.** Comparison of PNN with and without our weights/biases setting (RD denotes Random Division).

**Area Size**	**Computation Time**			**Overall Accuracy**	
	**Without RD**	**With RD**	**Ratio**	**Without RD**	**With RD**
**10 × 10**	1.0818	0.0231	46.8	94.8%	94.9%
**20 × 20**	4.0803	0.0386	105.7	95.5%	95.5%
**30 × 30**	22.4270	0.0751	298.6	96.3%	96.2%
**40 × 40**	58.1409	0.1125	516.8	95.9%	95.4%
